# GraphQA: protein model quality assessment using graph convolutional networks

**DOI:** 10.1093/bioinformatics/btaa714

**Published:** 2020-08-11

**Authors:** Federico Baldassarre, David Menéndez Hurtado, Arne Elofsson, Hossein Azizpour

**Affiliations:** Division of Robotics, Perception and Learning (RPL), KTH – Royal Institute of Technology, 10044 Stockholm, Sweden; Department of Intelligent Systems, Science for Life Laboratory, Stockholm University, Box 1031, 17121 Solna, Sweden; Department of Biochemistry and Biophysics, school of Electrical Engineering and Computer Science (EECS), Stockholm University, 10691 Stockholm, Sweden; Department of Intelligent Systems, Science for Life Laboratory, Stockholm University, Box 1031, 17121 Solna, Sweden; Department of Biochemistry and Biophysics, school of Electrical Engineering and Computer Science (EECS), Stockholm University, 10691 Stockholm, Sweden; Division of Robotics, Perception and Learning (RPL), KTH – Royal Institute of Technology, 10044 Stockholm, Sweden

## Abstract

**Motivation:**

Proteins are ubiquitous molecules whose function in biological processes is determined by their 3D structure. Experimental identification of a protein’s structure can be time-consuming, prohibitively expensive and not always possible. Alternatively, protein folding can be modeled using computational methods, which however are not guaranteed to always produce optimal results. GraphQA is a graph-based method to estimate the quality of protein models, that possesses favorable properties such as representation learning, explicit modeling of both sequential and 3D structure, geometric invariance and computational efficiency.

**Results:**

GraphQA performs similarly to state-of-the-art methods despite using a relatively low number of input features. In addition, the graph network structure provides an improvement over the architecture used in ProQ4 operating on the same input features. Finally, the individual contributions of GraphQA components are carefully evaluated.

**Availability and implementation:**

PyTorch implementation, datasets, experiments and link to an evaluation server are available through this GitHub repository: github.com/baldassarreFe/graphqa.

**Supplementary information:**

Supplementary data are available at *Bioinformatics* online.

## 1 Introduction

Protein molecules are predominantly present in biological forms, where they are responsible for most cellular functions. Therefore, understanding, predicting and modifying proteins in biological processes are essential for medical, pharmaceutical and genetic research. Such studies strongly depend on discovering mechanical and chemical properties of proteins through the determination of their structure.

At the high level, a protein molecule is a chain of hundreds of smaller molecules called amino acids. Identifying a protein’s amino-acid sequence is nowadays straightforward. However, the function of a protein is primarily determined by its 3D structure. Spatial folding can be determined experimentally, but the existing procedures are time consuming, prohibitively expensive and not always possible. Thus, several computational techniques were developed for protein structure prediction (Arnold *et al.*, 2006; Wang *et al.*, 2017; Xu, 2019). So far, no single method is always best, e.g. some proteins are best modeled by some specific method, also, computational methods often produce multiple outputs. Thus, candidate generation is generally followed by an evaluation step. This work focuses on quality assessment (QA) of computationally derived protein models (Lundstrom *et al.*, 2001; Won *et al.*, 2019).

Protein QA, also referred to as the estimation of model accuracy, estimates the quality of computational protein models in terms of divergence from their native structure. The downstream goal of QA is twofold: to find the best model in a pool of models and to refine a model based on its estimated local quality.

Computational protein folding and design have recently received attention from the machine learning community (AlQuraishi, 2019; Anand and Huang, 2018; Evans *et al.*, 2018; Jones and Kandathil, 2018; Ingraham *et al.*, 2019b; Wang *et al.*, 2017; Xu, 2019), while QA has yet to follow. This is despite the importance of QA for structural biology and the availability of standard datasets to benchmark machine learning techniques, such as the biannual CASP event (Moult *et al.*, 1999). The field of bioinformatics, on the other hand, has witnessed noticeable progress in QA for more than a decade: from earlier works using artificial neural networks (Wallner and Elofsson, 2006) or support vector machines (Ray *et al.*, 2012; Uziela *et al.*, 2016) to more recent works including MULTICOM (Hou *et al.*, 2019), SARTclust (submitted as group UOSHAN in CASP13) (Cheng *et al.*, 2019), ModFOLD7 (McGuffin *et al.*, 2019b), FaeNNz (Studer *et al.*, 2020) and those using deep learning techniques, such as 1D-CNNs, 3D-CNNs and LSTMs (Conover *et al.*, 2019; Derevyanko *et al.*, 2018; Hurtado *et al.*, 2018; Pagès *et al.*, 2018).

In this work, we tackle QA with graph convolutional networks (GCNs), which offer several desirable properties over previous methods. Through extensive experiments, we show GraphQA performs similarly to the state-of-the-art methods despite using a relatively low number of features. Particularly, in comparison to ProQ4 which uses the same set of input features, it provides a clear improvement in performance.

### 1.1 Related works

**Protein quality assessment** methods are evaluated in CASP (Moult *et al.*, 1995) since CASP7 (Cozzetto *et al.*, 2007). Current techniques can be divided into two categories: single-model methods which operate on a single protein model to estimate its quality (Wallner and Elofsson, 2003), and consensus methods that use consistency between several candidates to estimate their quality (Lundstrom *et al.*, 2001). Single-model methods are applicable to a single protein in isolation and in the recent CASP13 performed comparably to or better than consensus methods for the first time (Cheng *et al.*, 2019). Several recent single-model QA works are based on deep learning: 3DCNN and Ornate adopt a volumetric representation of proteins (Derevyanko *et al.*, 2018). Ornate improves 3DCNN by defining a canonical orientation (Pagès *et al.*, 2018). ProQ3D (Uziela *et al.*, 2017) uses a multilayer perceptron with carefully optimized residue descriptors from ProQ3 (Uziela *et al.*, 2016) as inputs. MULTICOM-NOVEL (Hou *et al.*, 2019) trains a 1DCNN with multitask learning to predict local and global scores. ProQ4 adopts a pretrained 1D-CNN that is fine-tuned in a Siamese configuration with a rank loss (Hurtado *et al.*, 2018) using exactly the same protein descriptors as used in this work. Other recent methods not based on deep learning include SBROD that uses ridge regression (Karasikov *et al.*, 2019), QMEANDisCo (Studer *et al.*, 2020) and VoroMQA that takes a statistical approach on atom-level contact area (Olechnovič *et al.*, 2013). VoroMQA and ProQ3D are among the top-performing methods of CASP13 (Won *et al.*, 2019) together with MULTICOM, ModFOLD7 (McGuffin *et al.*, 2019a) and SART (Cheng *et al.*, 2019), which use a large combination of protein predictors as inputs to the machine learning algorithm .

**Fig. 1.**
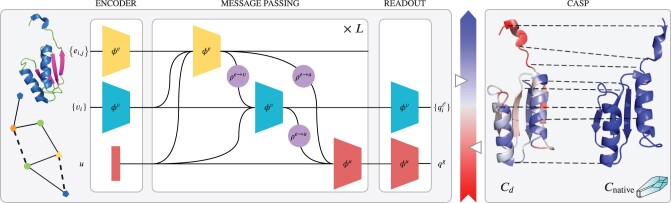
Protein QA. GraphQA predicts local and global scores from a protein’s graph using message passing between chemically bonded or spatially close residues. CASP QA algorithms score protein models by comparison with experimentally determined conformations

**Graph convolutional networks** bring the representation learning power of CNNs to graph data, and have been recently applied with success to multiple domains, e.g. physics (Gonzalez *et al.*, 2018), visual scene understanding (Narasimhan *et al.*, 2018) and natural language understanding (Kipf and Welling, 2017). In the chemistry domain, molecules can be naturally represented as graphs, and GCNs have been proven effective in several related tasks, including molecular representation learning (Duvenaud *et al.*, 2015), protein interface prediction (Fout *et al.*, 2017), chemical property prediction (Gilmer *et al.*, 2017; Li *et al.*, 2018a; Niepert *et al.*, 2016), drug–drug interaction (Zitnik *et al.*, 2018), drug–target interaction (Gao *et al.*, 2018), molecular optimization (Jin *et al.*, 2019) and generation of proteins, molecules and drugs (Ingraham *et al.*, 2019a; Li *et al.*, 2018b; Liu *et al.*, 2018; Simonovsky and Komodakis, 2018; You *et al.*, 2018). However, to the best of our knowledge, when we started this work, GCNs have never been applied to the problem of protein QA.

### 1.2 Contributions

- This work is the first to tackle QA with GCNs which bring several desirable properties over previous methods, including representation learning (3DCNN, Ornate), geometric invariance (VoroMQA, Ornate), sequence learning (ProQ4, AngularQA), explicit modeling of 3D structure (3DCNN, Ornate, VoroMQA) and computational efficiency.
- Thanks to these properties, a simple GCN setup performs similarly to state-of-the-art methods while using a relatively low number of features. Also, the graph network structure provides an improvement over the architecture used in ProQ4. This is demonstrated through extensive experiments on multiple datasets and scoring regimes.
- Novel representation techniques are used to explicitly reflect the sequential (residue separation) and 3D structure (angles, spatial distance and secondary structure) of proteins.
- Enabled by the use of GCN, we combine the optimization of local and global predictions for QA, improving over the performance of global-only or local-only scoring methods.
- Through an extensive set of ablation studies, the significance of different components of the method, including architecture, loss and features, are carefully analyzed.

## 2 Materials and methods

We start describing our method by arguing for the representation of protein molecules as graphs in learning tasks, then we define the problem of protein QA, and finally, we introduce the GraphQA architecture.

### 2.1 Protein representation as graphs

Proteins are large molecular structures that perform vital functions in all living organisms. At the chemical level, a protein consists of one or more chains of smaller molecules, which we interchangeably refer to as **residues** for their role in the chain, or as **amino acids** for their chemical composition. The sequence of residues $S=\{a_{i}\}$ that composes a protein represents its *primary structure*, where *a_i_* is one of the 22 amino acid types (20 natural ones, plus Selenocysteine and Pyrrolysine). The interactions between neighboring residues and the environment dictate how the chain will fold into complex spatial structures that represent the protein’s *secondary structure* and *tertiary structure*.

**Fig. 2.**
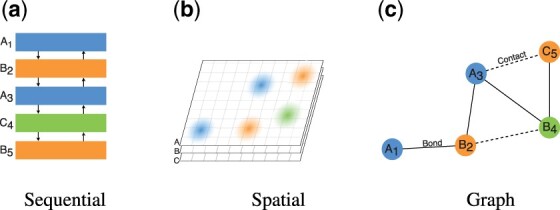
Protein representations for learning. Sequential representations for LSTM or 1D-CNN fail to represent spatial proximity of non-consecutive residues. Volumetric representations for 3D-CNN fail instead to capture sequence information and are not rotation invariant. Protein graphs explicitly represent both sequential and spatial structure, and are geometrically invariant by design

Therefore, a suitable representation for any learning task should reflect both the identity and sequence of the residues, i.e. the primary structure, and geometric information about the protein’s arrangement in space, i.e. its tertiary structure (Fig. 2). Some works use RNN or 1D-CNN to model proteins as a flat sequence of residues with the spatial structure potentially embedded in the handcrafted residue features (Conover *et al.*, 2019; Hurtado *et al.*, 2018). Other works model proteins’ spatial structure using volumes of atomic densities and 3D-CNNs, but do not explicitly use the sequential information contained in the residue chain (Derevyanko *et al.*, 2018; Pagès *et al.*, 2018). We argue that graph-based learning can explicitly model both the sequential and geometric structures of proteins. Moreover, it accommodates proteins of different lengths and spatial extent and is invariant to rotations and translations.

In the simplest form, a protein can be represented as a linear graph, where nodes represent amino acids and edges connect consecutive residues according to the primary structure. This set of edges, which represent the covalent **bonds** that form the protein backbone, can be extended to include the interactions between non-consecutive residues, e.g. through Van der Waals forces or hydrogen bonds, commonly denoted as **contacts**. By forming an edge between all pairs of residues that are within a chemically reasonable distance of each other, the graph becomes a rich representation of both the sequential and geometric structure of the protein (Fig. 2). To spatially locate residues and measure distances, we consider the coordinates of alpha carbons. We refer to this representation, composed of residues, bonds and contacts, as the **protein graph**:

$$P=(\{v_{i}\},\;\{e_{i,j}^{\text{bond}}||i-j|=1\}\cup \{e_{i,j}^{\text{contact}}||i-j|>1,\left\|C_{i}-C_{j}\right\|\leq d_{\text{max}}\}),$$

where i,j=1,…,|S| are residue indices, $C=\{{\left(x,y,z\right)}_{i}\}$ are the coordinates of each residue’s alpha carbon, representing the protein’s **conformation**, and $d_{\text{max}}$ is a cutoff distance for contacts.

With the protein’s structure encoded in the graph, additional residue and relationship features can be encoded as nodes and edges attributes, $v_{i}$ and $e_{i,j}$ respectively. Section 3.2 describes, in detail, an attribution that preserves the sequence information and 3D geometry while remaining invariant to rotation.

### 2.2 Protein quality assessment

Experimental identification of a protein’s **native structure** can be time consuming and prohibitively expensive. Alternatively, computational folding methods are used to generate **decoy** conformations for a specific **target** protein. Since no single method is consistently best, a QA step is used to identify the conformations *C^d^* that most correctly represent the native structure.

If the native structure $C^{\text{native}}$ is experimentally determined, the quality of a decoy can be measured by comparing the decoy with the native structure. In the CASP challenge, decoys submitted for a target are scored against the unreleased native structure. Some QA methods compute global (per decoy) scores, which can be used for ranking and represent the principal factor for CASP, while others produce local (per residue) scores which help identify incorrect parts of a decoy (Uziela *et al.*, 2018).

In most scenarios, however, the native structure is not available, and quality must be estimated based on physical and chemical properties of the decoy, e.g. in drug development, it would be unpractical to synthesize samples of novel proteins and researchers rely on computational folding and QA instead.

Here, we introduce GraphQA, a graph-based neural network that learns to predict global and local QA scores, with a relatively low number of features and minimal model engineering, using existing datasets of scored proteins. At the residue level, GraphQA is trained to output the Local Distance Difference Test (Mariani *et al.*, 2013) and the Contact Area Difference (Olechnovič *et al.*, 2013) scores. For a residue *i*, we denote them as: $q_{i}^{\ell }:=[{\text{LDDT}}_{i},{\text{CAD}}_{i}]$.

At the decoy level, GraphQA is trained to output widely used scores: Global Distance Test Total Score, which is the official CASP score for protein-level QA, Global Distance Test High Accuracy (Zemla, 2003), TM-score (Zhang and Skolnick, 2004) and the global versions of LDDT and CAD. Together, we denote them as: $q^{g}:=[\text{GDT}\_\text{TS},\text{GDT}\_\text{HA},\text{TM},\text{LDDT},\text{CAD}]$.

With ${\text{GraphQA}}_{i}^{\ell }(P)$ and ${\text{GraphQA}}^{g}(P)$ denoting the network’s local and global predictions for an input P, the learning objective is to minimize the following Mean Squared Error (MSE) losses:

(1) $$\begin{array}{cc} & \ell_{\ell }=\sum_{i}^{\left|S\right|}{\left[{\text{GraphQA}}_{i}^{\ell }(P)-q_{i}^{\ell }\right]}^{2},\\ & \ell_{g}={\left[{\text{GraphQA}}^{g}(P)-q^{g}\right]}^{2}. \end{array}$$

Note that, for the sole purpose of sorting decoy according to ground-truth quality, training with a ranking loss would be sufficient (Derevyanko *et al.*, 2018). Instead, MSE forces the output to match the quality score, which is a harder objective, but results in a network can be more easily inspected and possibly used to improve existing folding methods in an end-to-end fashion (Section 4.3).

### 2.3 GraphQA architecture

GraphQA is a GCN that operates on protein graphs using the message-passing algorithm described by Battaglia *et al.* (2018). The building block of GraphQA, a graph layer, takes a protein graph as input (with an additional global feature u), and performs the following propagation steps to output a graph with updated node/edge/global features and unchanged structure:

$$\begin{array}{ll} e\prime_{i,j}=\phi^{e}\left(e_{i,j},v_{i},v_{j},u\right) & \text{Update edges}\\ \bar{e}\prime_{i}=\rho^{e\to v}\left(\left\{e\prime_{j,i}\right\}\right) & \text{Aggregate edges}\\ v\prime_{i}=\phi^{v}\left(\bar{e}\prime_{i},v_{i},u\right) & \text{Update nodes}\\ \bar{e}\prime =\rho^{e\to u}\left(\left\{e\prime_{i,j}\right\}\right) & \text{Aggregate all edges}\\ \bar{v}\prime =\rho^{v\to u}\left(\left\{v\prime_{i}\right\}\right) & \text{Aggregate all nodes}\\ u\prime =\phi^{u}\left(\bar{e}\prime ,\bar{v}\prime ,u\right) & \text{Update global features} \end{array}$$

where ϕ represent three update functions that transform nodes/edges/global features (e.g. a MLP), and *ρ* represent three pooling functions that aggregate features at various levels (e.g. sum or mean).

Similarly to CNNs, multiple graph layers are stacked to propagate local information to increasingly larger neighborhoods, i.e. receptive field. This enables the network to learn quality-related features at multiple scales: secondary structures in the first layers, e.g. *α*-helices and *β*-sheets, and larger structures in deeper layers e.g. domain structures and arrangements.

The GraphQA architecture is conceptually divided into three stages (Fig. 1). At the input, the **encoder** increases the node and edge features’ dimensions through 2× (Linear-Dropout-ReLU) transformation and adds a global bias. Then, at its core, *L* **message-passing** layers operate on the encoded graph, leveraging its structure to propagate and aggregate information. The update functions ϕ consist of Linear-Dropout-ReLU transformations, with the size of the linear layers progressively decreasing. We use average pooling for the aggregation functions *ρ*, since preliminary experiments with max/sum pooling performed poorly. Finally, the **readout** layer outputs local and global quality scores by applying a Linear-Sigmoid operation to the latest node and global features, respectively.

## 3 Experiments

### 3.1 Datasets

Following the common practice in QA, we use the data from past years’ editions of CASP, encompassing several targets with multiple scored decoys each. From CASP 9–12, we assemble a dataset of 85k decoys ${\left(P,\{q_{i}^{\ell }\},q^{g}\right)}^{t,d}$, which we randomly split into a training set (∼270 targets) and a validation set for hyperparameter optimization (∼50 targets). These targets are also used for the extensive ablation studies described in Section 4.2 and in Supplementary Appendix S4. To compare GraphQA against other top-scoring methods, we collect the ∼14k stage-2 decoys across 72 targets of CASP 13 as a test set. We obtain ground-truth scores for training and evaluation by comparing each decoy with the released native structure. Further details on data collection and processing are available in Supplementary Appendix S2.1.

### 3.2 Features

**Node features** The node attributes $v_{i}$ of a protein graph P represent the identity, statistical and structural features of the *i*th residue. We encode the residue identity using a one-of-22 encoding of the corresponding amino acid. Following Hurtado *et al.* (2018), we also add residue-level statistics computed using Multiple Sequence Alignment (MSA) (Rost *et al.*, 1994), namely *self-information* and *partial entropy*, each described by a 23-dimensional vector. Finally, we add a 14-dimensional vector of spatial features including the dihedral angles, surface accessibility and secondary structure type as determined by DSSP (Kabsch and Sander, 1983).

**Edge features** An edge represents either a contact or a bond between two residues *i* and *j* w.r.t. to the conformation $C=\{{\left(x,y,z\right)}_{i}\}$. An edge always exists between two consecutive residues, while non-consecutive residues are only connected if $||C_{i}-C_{j}||<d_{\text{max}}$, with $d_{\text{max}}$ optimized on the validation set. We further enrich this connectivity structure by encoding spatial and sequential distances as an 8D feature vector $e_{i,j}$. Spatial distance is encoded using a radial basis function $\text{exp}(-d_{i,j}^{2}/\sigma )$, with *σ* determined on the validation set. Sequential distance is defined as the number of amino acids between the two residues in the sequence and expressed using a **separation encoding**, i.e. a one-hot encoding of the separation |i−j| according to the classes {0,1,2,3,4,5:10,>10}.

### 3.3 Optimization and hyperparameter search

The MSE losses in Equation 1 are weighted as $\ell_{\mathrm{tot}}=\lambda_{\ell }\ell_{\ell }+\lambda_{g}\ell_{g}$ and minimized using Adam Optimizer (Kingma and Ba, 2014) with *L*_2_ regularization. GraphQA is significantly faster to train than LSTM or 3D-CNN methods, e.g. 35 epochs take ∼2 hours on one NVIDIA 2080Ti GPU with batches of 200 graphs, thus allowing for an extensive hyperparameter search. Supplementary Appendix S3.2 reports search space, optimization procedure and the parameters of the model with highest $R_{\text{target}}$ on the validation set.

## 4 Evaluation

We compare GraphQA with other single-model methods which are top-performing in CASP13 and/or represent a relevant approach for QA. ProQ3D computes fixed-size statistical descriptions of the decoys in CASP 9-10, including Rosetta energy terms, which are then used to train a Multilayer Perceptron on quality scores (Uziela *et al.*, 2017). In ProQ4, a 1D-CNN is trained to predict LDDT scores from a vectorized representation of protein sequences, a global score is then obtained by averaging over all residues (Hurtado *et al.*, 2018). ProQ4 is pretrained on a large dataset of protein secondary structures and then fine-tuned on CASP 9-10 using a Siamese configuration to improve ranking performances. 3DCNN (group name: LamoureuxLab) trains a CNN on a three-dimensional representation of atomic densities to rank the decoys in CASP 7–10 according to their GDT_TS scores (Derevyanko *et al.*, 2018). Notably, no additional feature is used other than atomic structure and type, however, the fixed-size volumetric representation of this method is sensitive to rotations and does not scale well with protein size. Ornate (group name: 3DCNN) applies a similar 3D approach to predict local CAD scores and achieves rotation invariance by specifying a canonical residue-centered orientation (Pagès *et al.*, 2018). Although optimized for local scoring, the average of the predicted scores is shown to correlate well with GDT_TS. AngularQA, feeds a sequence-like representation of the protein structures from 3DRobot and CASP 9–12 to an LSTM to predict GDT_TS scores (Conover *et al.*, 2019). VoroMQA is a statistical potential method that represents an alternative to the other machine learning-based methods (Olechnovič *et al.*, 2013). SART (group name: SASHAN) combines statistical- and consistency-based terms to predict global and local scores (Cheng *et al.*, 2019).

### 4.1 Results

We evaluate all methods on a common subset of 72 CASP13 targets for which official submissions are publicly available (list in Supplementary Appendix S6).

**Global metrics** For the main experiments, we restrict the evaluation of global performances to GDT_TS, since it is the official score for CASP and all participants are expected to submit predictions for GDT_TS. Further results for GDT_HA and TM-score are available in the Supplementary Material. For each QA method, we consider the predicted and ground-truth scores and compute: Root Mean Squared Error (RMSE), Pearson correlation coefficient computed across all decoys of all targets (*R*), Pearson correlation coefficient computed on a per-target basis and then averaged over all targets ($R_{\text{target}}$), *z*-score of the top-scoring decoy of each target and averaged across targets (*z*), and the minimum difference between the true score of the best decoy and the true scores of the five highest-ranking decoys for each target averaged over targets (${\text{FRL}}_{5}$).

**Local metrics** GraphQA predicts LDDT and CAD scores, to enable a valid comparison with the local scores predicted by other methods, we compute the absolute Spearman correlation coefficient between predicted and ground-truth scores. Specifically, we compute: Spearman correlation coefficient across all residues of all decoys of all targets (*ρ*), and Spearman correlation coefficient on a per-decoy basis and then averaged over all decoys of all targets ($\rho_{\text{decoy}}$). Of these, we focus on $R_{\text{target}}$ and $\rho_{\text{decoy}}$, which, respectively, measure the ability to rank decoys by quality and to distinguish the correctly predicted parts of a model from those that need improvement. See Supplementary Appendix S5 for more details and definitions.

**Fig. 3.**
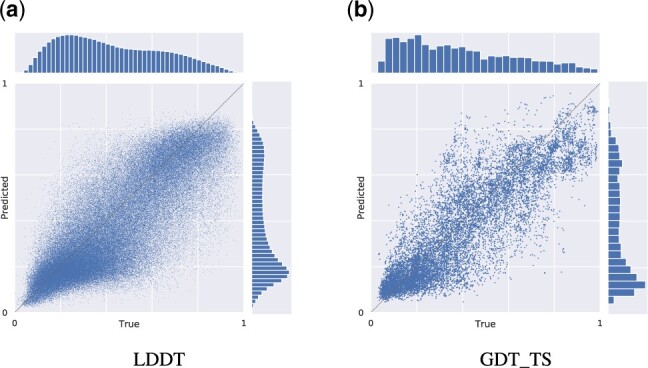
Joint plots of LDDT and GDT_TS scores on CASP13. The marginal plots show the distribution of true versus predicted scores

Table 1 compares the performances of GraphQA and other state-of-the-art single-model methods on GDT_TS predictions for CASP13, while Figure 3 contains a graphical representation of true versus predicted GDT_TS and LDDT scores for all targets in CASP13. At the global level, a noticeably higher $R_{\text{target}}$ metric indicates that GraphQA is more capable than other state-of-the-art single-model QA methods at ranking decoys of a target based on their overall quality. The 95% confidence interval for $R_{\text{target}}$ computed using the Fisher *r*-to-*z* method is [.772,.786]. Additional results for our method are reported in Supplementary Appendix S6.

**Table 1.** CASP13 global quality assessment

Method	RMSE ↓	R↑	$R_{\text{target}}↑$	*z* ↑	${\text{FRL}}_{5}↓$
**GraphQA**	**0.130**	0.855	**0.779**	**1.274**	0.030
ModFOLD7_rank	0.156	**0.872**	0.742	1.063	**0.023**
**GraphQA-RAW**	0.158	0.769	0.720	0.962	0.051
ProQ4	0.176	0.698	0.664	0.870	0.028
FaeNNz	0.141	0.803	0.661	0.865	0.032
ProQ3D	0.146	0.802	0.637	0.815	0.024
VoroMQA-A	0.208	0.657	0.555	0.755	0.041
Ornate	0.205	0.478	0.490	0.535	0.058
PLU-AngularQA	0.193	0.574	0.421	0.425	0.049
MULTICOM_CLUSTER	0.103	0.908	0.839	1.112	0.025
UOSHAN	0.090	0.925	0.865	1.122	0.030

*Note*: RMSE, Pearson corr., *z*-score and top-5 rank loss w.r.t. GDT_TS scores (normalized in [0,1]). Top: single-models methods sorted by $R_{\text{target}}$. At the bottom: consensus methods for context. Best results in bold.

Evaluation metrics for local quality predictions w.r.t. ground-truth CAD and LDDT scores are reported in Table 2. At the local level, our method proves to be on a par with best-performing methods, demonstrating the ability to evaluate quality at the residue level and distinguishing correctly predicted parts of the protein chain. Interestingly, GraphQA and ProQ4 use the same input features and they both co-optimize for local and global predictions, but the former achieves much better performances than the latter. We argue that the graph-based architecture allows GraphQA to capture more complex and long-range dependencies between residues than the Siamese 1D-CNN used in ProQ4 (Fig. 6).

**Table 2.** CASP13 local quality assessment

	CAD		LDDT	
Method	|ρ|↑	$|\rho_{\text{decoy}}|↑$	|ρ|↑	$|\rho_{\text{decoy}}|↑$
ProQ3D-lDDT	0.611	0.380	0.754	**0.543**
**GraphQA**	**0.664**	**0.423**	**0.797**	0.527
FaeNNz	0.648	0.361	0.794	0.523
ModFOLD7	0.523	0.336	0.678	0.501
ProQ3D-CAD	0.638	0.415	0.728	0.499
**GraphQA-RAW**	0.613	0.385	0.730	0.497
ProQ4	0.549	0.326	0.677	0.474
3DCNN	0.539	0.298	0.688	0.431
VoroMQA-A	0.499	0.285	0.600	0.412
Ornate	0.415	0.286	0.462	0.373
UOSHAN	0.517	0.317	0.688	0.488
ModFOLDclust2	0.486	0.338	0.641	0.512

*Note*: Global and per-decoy absolute Spearman corr. are reported w.r.t. ground-truth CAD and LDDT. Above the line: single-models methods sorted by LDDT $\left|\rho_{\text{decoy}}\right|$, consensus methods below. Best results

As shown in our ablation studies, hand-engineered features like MSA and DSSP contribute to the performances of GraphQA (Fig. 5), yet we wish to prove that our method can learn directly from raw data. GraphQA-RAW is a variant that relies uniquely on the one-hot encoding of amino acid identity, similarly to how 3D-CNN and Ornate use atomic features only. The results for GraphQA-RAW show that the graph representation and the GCN architecture are effective at automatically extracting features that are almost as expressive as the hand-engineered features used by the full GraphQA.

### 4.2 Ablation studies

Here, we analyze how various components of GraphQA contribute to the final performance, ranging from optimization and architectural choices to protein feature selection. Unless stated otherwise, all ablation studies follow the training procedure described in Section 3.3 for a lower number of epochs. We report results on CASP 11 as mean and std dev of 10 runs.

**Local and global co-optimization** We investigate the interplay between local and global predictions, specifically whether co-optimizing for both is beneficial or detrimental. At the global level, models trained to predict only global scores achieve a global RMSE of 0.129±.007, whereas models trained to predict both local and global scores obtain 0.117±.006, suggesting that local scores can provide additional information and help the assessment of global quality. At the local level instead, co-optimization does not seem to improve performances: models trained uniquely on local scores achieve a local RMSE of 0.121±.002, while models trained to predict both obtain 0.123±.004.

**Connectivity and architecture** In this study, we test the combined effects of the depth of the network *L* and the cutoff value $d_{\text{max}}$. Every additional message-passing layer allows to aggregate information from a larger neighborhood, effectively extending the receptive field at the readout. Also, the num. of included contacts affects graph connectivity and message propagation: low $d_{\text{max}}$ correspond to low average degree and long shortest paths between any two residues, and vice versa (Supplementary Appendix S2.2).

Thus, an architecture that operates on sparsely connected graphs will require more message-passing layers to achieve the same holistic view of a shallow network operating on denser representations. However, this trade-off is only properly exposed if $u,\phi^{u},\rho^{u}$ are removed from the architecture. In fact, a global pathway creates a shortcut that connects all nodes in the graph and sidesteps the limitations of shallow networks. With the global pathway disabled, global predictions are computed in the readout layer by aggregating node features from the last MP layer.

**Fig. 4.**
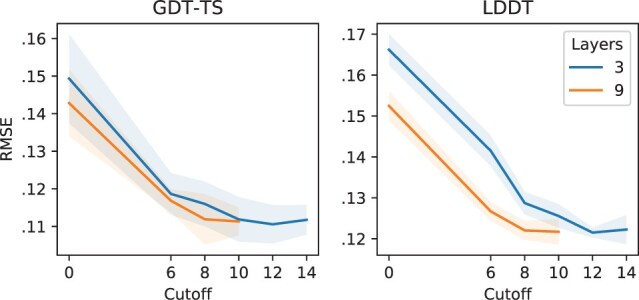
Trade-off between the number of message-passing layers and the connectivity of the protein graph (CASP11)

Figure 4 reports the RMSE obtained by networks of different depths with no global path, operating on protein graphs constructed with different cutoff values. As expected, the shallow 3-layer architecture requires more densely connected inputs to achieve the same performances of the 9-layer network. Surprisingly, local predictions seem to be more affected by these factors than global predictions, suggesting that a large receptive field is important even for local scores.

**Fig. 5.**
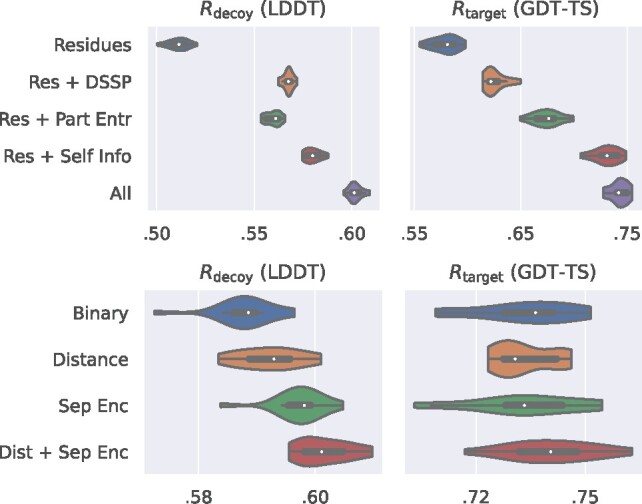
Ablation study of node (top) and edge (bottom) features (validation results on CASP 11). All node features improve both local and global scoring. DSSP features are marginally more relevant for LDDT. Richer edge features benefit LDDT predictions the most, while bringing little improvement to GDT_TS

**Node and edge features** We evaluate the impact of node and edge features on the overall prediction performances (Fig. 5). For the nodes, we use the amino acid identity as a minimal representation and combine it with: (i) DSSP features, (ii) partial entropy, (iii) self-information, (iv) both DSSP and MSA features. All features improve both local and global scoring, with DSSP features being marginally more relevant for LDDT. For the edges, we evaluate the effect of having either: (i) a binary indicator of bond/contact, (ii) geometric features, i.e. the Euclidean distance between residues, (iii) sequential features, i.e. the categorical encoding of the separation between residues, (iv) both distance and separation encoding. Progressively richer edge features seem to be benefit LDDT predictions, while little improvement can be seen at the global level.

### 4.3 Visualization and explainability

**Fig. 6.**
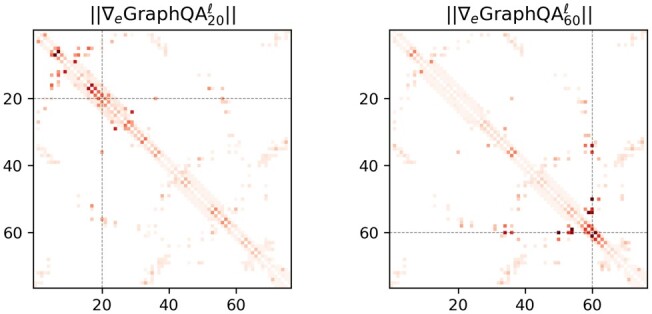
Gradient magnitude of predicted LDDT score w.r.t. the edges of the input graph (T0773). In the edge matrix, a darker red indicates a higher magnitude. The attributions for residue 20 (left) and 60 (right) reveal the long-range dependencies between residues captured by GraphQA

Since GraphQA is fully differentiable, the trained model can be used to explain the factors that influenced a low score and thereby provide potentially useful feedback for protein structure refinement. A simple approach for explaining predictions of a differentiable function f(x) is Sensitivity Analysis (Baehrens *et al.*, 2010), which uses $\left\|\nabla_{x}f\right\|$ to measure how variations in the input affect the output. In Figure 6, we consider the LDDT score predicted for two different residues and compute the magnitude of the gradients w.r.t. the edges of the graph. Thanks to its GCN architecture, GraphQA is able to capture quality-related dependencies not only in the neighborhood of the selected residues, but also further apart in the sequence.

We further probe the feasibility of structure refinement with a simple experiment and leave elaborate experiments as future work. If the network has learned a meaningful scoring function, then the gradient of the score w.r.t. the contact distances should aim in the direction of the native structure. Considering all decoys of all targets in CASP 11, we obtain an average cosine similarity $\text{cos}\;\left(\partial {\text{GraphQA}}^{g}/\partial d,\;d_{\text{decoy}}-d_{\text{native}}\right)$ of 0.14±.08, which suggests that gradients can be used as a coarse feedback for end-to-end protein structure prediction.

## 5 Conclusion

We applied GCNs to the important problem of protein QA. Since proteins are naturally represented as graphs, GCN allowed us to collect the individual benefits of the previous QA methods including representation learning, geometric invariance, explicit modeling of sequential and 3D structure, simultaneous local and global scoring, and computational efficiency. Thanks to these benefits, and through an extensive set of experiments, we demonstrated similar performance levels compared to the state-of-the-art in single-model QA using various metrics and datasets. This is achieved using relatively low number of features. We further analyzed the results via thorough ablation and qualitative studies. It is important to note that our tests were conducted offline while the other methods’ performance are taken from the blind test of CASP13 challenge. Thus, a fair comparison will only be possible when the results of CASP14 become available.

Finally, we believe that richer geometric representations, e.g. including relative rotations, and atom-level graphs could represent an interesting future direction for learning-based QA.

## Funding

This work was supported by Swedish E-science Research Council, the Swedish National Infrastructure for Computing, and the Swedish Research Council (Vetenskapsrådet). Project 2017-04609 to HA and 2016-03798 to AE.

*Conflict of Interest*: none declared.

## Supplementary Material

btaa714_Supplementary_Data
