# Supplementary material for: GraphQA: protein model quality assessment using graph convolutional networks
Source: Bioinformatics. 2020 Aug 11;37(3):360–6. doi: 10.1093/bioinformatics/btaa714 (PMC8058777; doi:10.1093/bioinformatics/btaa714)
Supplement: btaa714_Supplementary_Data [file btaa714_supplementary_data.pdf]

GraphQA: Supplementary Material

S1 Protein Quality Assessment

For the interested reader, we describe here in more detail how the Global Distance Test Total Score (Zemla, 2003) and the Local Distance Difference Test (Mariani *et al.*, 2013) are computed. Furthermore, we provide an intuition over what the benefits and downsides of each method are and motivate why a better quality assessment should consider both a global measure and a local measure.

Global Distance Test Total Score (GDT_TS) Global Distance Test Total Score (GDT_TS) is a global-level score obtained by first superimposing the structure of a decoy to the experimental structure using an alignment heuristic, and then computing the fraction of residues whose position is within a certain distance from the corresponding residue in the native structure (figure S1). This percentage is computed at different thresholds and then averaged to produce a score in the range $[0, 100]$, which we rescale between 0 and 1 (table S1).

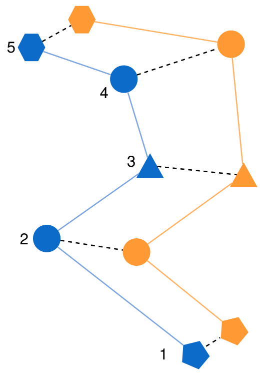

Fig. S1: Description of GDT_TS

Local Distance Difference Test (LDDT) Local Distance Difference Test (LDDT), is a residue-level score that does not require alignment of the structures and compares instead the local neighborhood of every residue, in the decoy and in the native structure. If we define the neighborhood of a residue as the set of its contacts, i.e. the set of other residues that lie within a certain distance from it, we can express the quality of that residue as the percentage of contacts that it shares with the corresponding residue in the native structure.

Table S1. Cuttoffs used in lDDT

i	$\|\|C_i^d - C_i^{\text{native}}\|\|$	< 1	< 2	< 5	< 10
1	0.6 Å	x	x	x	x
2	1.2 Å		x	x	x
3	1.9 Å		x	x	x
4	2.5 Å			x	x
5	6.3 Å				x
		20%	60%	80%	100%

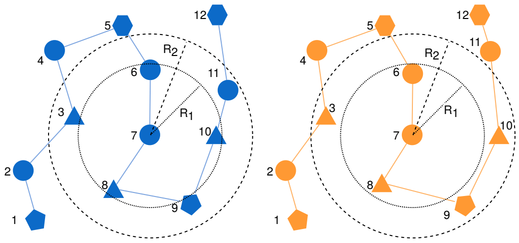

Fig. S2: Example of LDDT scoring for residue 7: the residues within a radius R_1 are $\{6, 8, 10\}$ the native structure (left) and $\{6, 8\}$ for the decoy (right); at a radius R_2 we have $\{3, 6, 8, 9, 10, 11\}$ the native structure (left) and $\{3, 6, 8, 9, 10\}$ for the decoy (right).

S2 Datasets, software and statistics

S2.1 Datasets and software

We consider all decoys of all target included in CASP 9-13, excluding targets that have been canceled by the organizers (table S2). For each target, we consider the native models available on the CASP website as the ground-truth tertiary structure.

Table S2. Datasets from previous CASP editions

Dataset	Targets	Models	Usage
CASP9	106	33627	Train/Val
CASP10	100	24525	
CASP11	82	16012	
CASP12	39	6689	
CASP13	72	14616	Test

The following auxiliary tools and programs are used to preprocess the data downloaded from CASP:

- We compare each decoy to its corresponding native structure and obtain the ground-truth scores that we use to train and evaluate our method:
  - TM-score (`https://zhanglab.ccmb.med.umich.edu/TM-score`) to evaluate TM-score, GDT_TS, GDT_HA,
  - lDDT (`https://swissmodel.expasy.org/lddt`) to evaluate global and local LDDT scores,
  - Voronota (`https://bitbucket.org/kliment/voronota`) to evaluate global and local CAD scores.
- For each target sequence, we run the Jackhmmer (`http://hmmer.org/documentation.html`) tool for multiple-sequence alignment against the Uniref50 dataset.
- For each decoy, we extract DSSP features to describe the secondary structure using the DSSP software (`https://swift.cmbi.umcn.nl/gv/dssp/index.html`).

S2.2 Protein graphs statistics

The cutoff value d_{max} determines which edges are included in the graph and, consequentially, its connectivity. A low cutoff implies a sparsely connected graph, with few edges and long paths between nodes. A higher cutoff yields a denser graph with more edges and shorter paths.

In figure S3 we report some statistics about number of edges, average degree and average shortest paths, evaluated at different cutoff values on 1700 decoys from CASP 11.

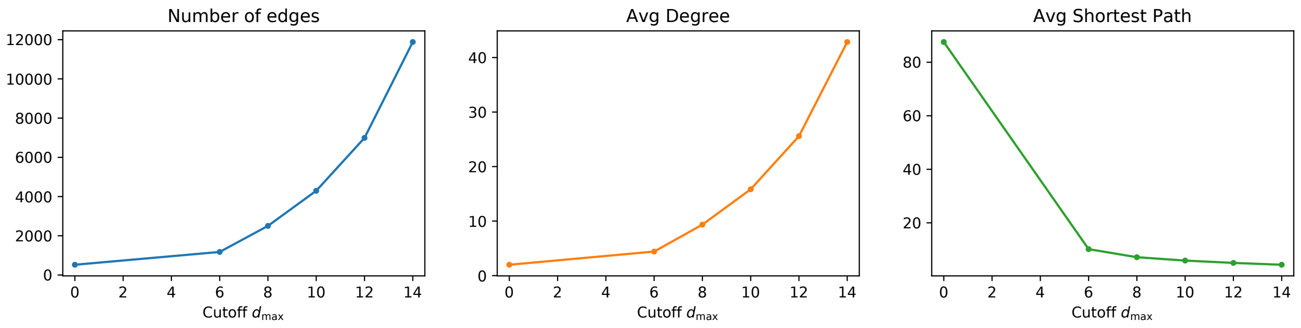

Fig. S3: Number of edges, average degree, and average shortest paths at different cutoff values (sample size: 1700 decoys from CASP 11)

S3 GraphQA architecture

In this section, we illustrate in more detail the structure of the GraphQA architecture, as well as the hyperparameter space that was explored to optimize performances on the validation set.

S3.1 Message-passing layers

Within GraphQA, a protein structure is represented as a graph whose nodes correspond to residues and whose edges connect interacting pairs of amino acids. At the input, the features of the i-th residue are encoded in a node feature vector $\boldsymbol{v}_i$. Similarly, the features of the pairwise interaction between residues i and j are encoded in an edge feature vector $\boldsymbol{e}_{i,j}$. A global bias term $\boldsymbol{u}$ is also added to represent information that is not localized to any specific node/edge of the graph.

With this graph representation, one layer of message passing performs the following updates.

1. For every edge $i \rightarrow j$, the edge feature vector is updated using a function ϕ^e of adjacent nodes $\boldsymbol{v}_i$ and $\boldsymbol{v}_j$, of the edge itself $\boldsymbol{e}_{i,j}$ and of the global attribute $\boldsymbol{u}$:
$$\boldsymbol{e}'_{i,j} = \phi^e\left(\boldsymbol{e}_{i,j}, \boldsymbol{v}_i, \boldsymbol{v}_j, \boldsymbol{u}\right)$$
2. For every node i, features from incident edges $\{\, \boldsymbol{e}'_{j,i} \,\}$ are aggregated using a pooling function $\rho^{e\rightarrow v}$:
$$\bar{\boldsymbol{e}}'_i = \rho^{e\rightarrow v}\left(\{\, \boldsymbol{e}'_{j,i} \,\}\right)$$
3. For every node i, the node feature vector is updated using a function ϕ^v of aggregated incident edges $\bar{\boldsymbol{e}}'_i$, of the node itself $\boldsymbol{v}_i$ and of the global attribute $\boldsymbol{u}$:
$$\boldsymbol{v}'_i = \phi^v\left(\bar{\boldsymbol{e}}'_i, \boldsymbol{v}_i, \boldsymbol{u}\right)$$
4. All edges are aggregated using a pooling function $\rho^{e\rightarrow u}$:
$$\bar{\boldsymbol{e}}' = \rho^{e\rightarrow u}\left(\{\, \boldsymbol{e}'_{i,j} \,\}\right)$$
5. All nodes are aggregated using a pooling function $\rho^{v\rightarrow u}$:
$$\bar{\boldsymbol{v}}' = \rho^{v\rightarrow u}\left(\{\, \boldsymbol{v}'_i \,\}\right)$$
6. The global feature vector is updated using a function ϕ^u of the aggregated edges $\bar{\boldsymbol{e}}'$, of the aggregated nodes $\bar{\boldsymbol{v}}'$ and of the global attribute $\boldsymbol{u}$:
$$\boldsymbol{u}' = \phi^u\left(\bar{\boldsymbol{e}}', \bar{\boldsymbol{v}}', \boldsymbol{u}\right)$$

In GraphQA, all intermediate updates are implemented as Linear-Dropout-ReLU functions, and all aggregation functions use average pooling. The encoder and readout layers do not make use of message passing, effectively processing every node/edge in isolation. Message passing is instead enabled for the core layers of the network and enables GraphQA to process information within progressively expanding neighborhoods.

The number of neurons in the core message-passing layers decreases from the input to the output. Specifically it follows a linear interpolation between the input and output numbers reported below, rounded to the closest power of two. In preliminary experiments, we noticed that a progressive increase of the number of layers results in convergence issues, which is in contrast to the practice of increasing the number of channels in Convolutional Neural Networks.

S3.2 Hyperparameter optimization

GraphQA has a low memory footprint (we can process mini-batches of 200 graphs on a 12GB GPU card) and is computationally efficient (training takes 2 hours using a single GPU), which allows for extensive hyperparameter search and ablation studies. Rather than performing a full-range grid search over the whole space, we perform a "guided" parameter search. First we perform a few exploratory runs, then we decide to focus on the most promising combinations of parameters and skip others. For example, in some initial experiments we tried *max pooling* as an aggregation function ρ for message-passing layers, but it performed poorly on the validation set and we decided to exclude it. Repeating this process of trial and elimination allows us to prune unpromising regions of the search space.

The final model is chosen to be the one with the highest R_{target} on the validation set. The following considerations were made:

- The range of values for d_{max} is chosen according to the average distance between alpha carbons of two consecutive residues, which is approximately ~ 5Å, and a distance ~ 10Å after which residue-residue interactions are negligible.
- The values for σ are chosen so that the RBF encoding of the edge length is approximately linear around ~ 5Å.
- The values for L are chosen to approximately match the average length of the shortest paths in the protein graphs at different cutoffs.
- In addition to what described in section 2.3, we also tested an architecture with BatchNorm layers between the Dropout and ReLU operations, but apart from a significant slowdown we did not notice any improvement.

Table S3. Hyper parameter space and best values

**Hyper parameter**	**Values**	**Best**
MP Layers L	3, 4, 5, 6, 7, 8, 9	6
MP input size $\boldsymbol{e}$	32, 64, 128	128
MP input size $\boldsymbol{v}$	64, 128, 256, 512	512
MP input size $\boldsymbol{u}$	64, 128, 256, 512	512
MP output size $\boldsymbol{e}$	8, 12, 16, 32	16
MP output size $\boldsymbol{v}$	8, 16, 32, 64	64
MP output size $\boldsymbol{u}$	8, 12, 16, 32	32
Cutoff d_{max}	6, 8, 10, 12	8
Sigma σ	10, 15, 20	15
Dropout rate	0, 0.1 0.2, 0.3, 0.4	0.2
Learning rate	$10^{-2}, 10^{-3}$	10^{-3}
Weight decay	$10^{-4}, 10^{-5}$	10^{-5}
Local weight λ^{ℓ}	1, 5, 10	1
Global weight λ^{g}	1, 5, 10	1

For some of the final hyperparameters, the best value happens to be at the boundary of the search space, e.g. for the sizes of the message-passing layers. In those cases, we decided to stop the search and not extend the range further for practical reasons: i) those parameters control the number of weights in the network, the larger the network, the heavier the risk of overfit, ii) increasing some of those values to 768 or 1024 would have slowed down/increased the memory footprint too much, iii) by observing the trend from smaller values to larger values we noticed diminishing returns and assumed that no significant improvement could be achieved by searching further in that direction.

S4 Additional Studies

To complement the analysis reported in the main text, we perform additional studies on the effect of feature representation and on the generalization ability of the trained model.

S4.1 Distance and separation encoding

The feature vectors associated to the edge of the graph represent two types of distances between residues, namely spatial distance and separation in the sequence. In this study we evaluate the effect of different representations on validation performances.

Spatial distance is the physical distance between ammino acids, measured as the euclidean distance between their β carbon atoms. We consider three possible encodings for this distance:

- **Absent:** spatial distance is not provided as input;
- **Scalar:** spatial distance is provided as a raw scalar value (in Ångstrom);
- **RBF:** spatial distance is encoded using 32 RBF kernels, with unit variance, equally spaced between 0 and 20.

Figure S4 reports the aggregated performances on CASP 11 of ten runs for each of the above. The rich representation of the RBF kernels seem to improve both LDDT and GDT_TS scoring performances, even though the effect is rather limited.

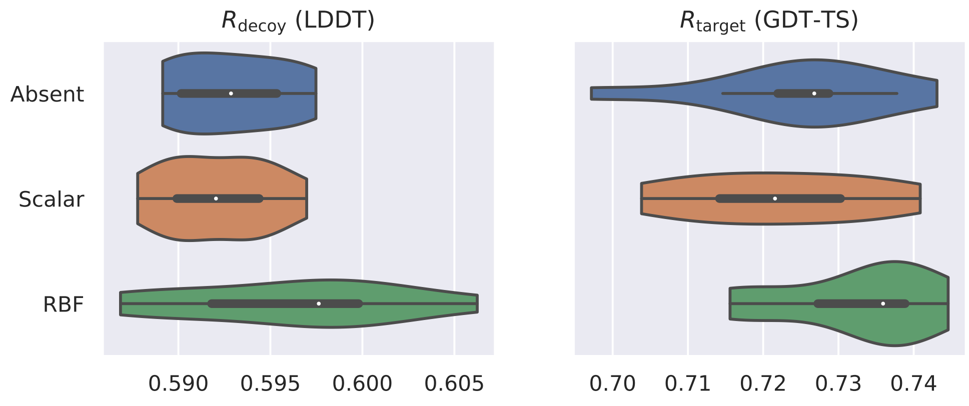

Fig. S4: Spatial distance: absent, encoded as a scalar, encoded using RBF kernels.

Separation is the number of residues between to amino acids in the sequence, we consider three possible encodings:

- **Absent:** sequential separation is not provided as input;
- **Scalar:** sequential separation is provided as a raw scalar value (positive integer);
- **Categorical:** sequential separation is encoded as a one-hot categorical variable, according to the classes $\{0, 1, 2, 3, 4, 5:10, >10\}$, which are based on typical interaction patterns within a peptidic chain.

Figure S5 reports the aggregated performances on CASP 11 of ten runs for each of the above. For local scoring, the choice of encoding plays little difference as long as separation is present in the input. On the other hand, the choice of categorical encoding over scalar encoding results in higher global scoring performance.

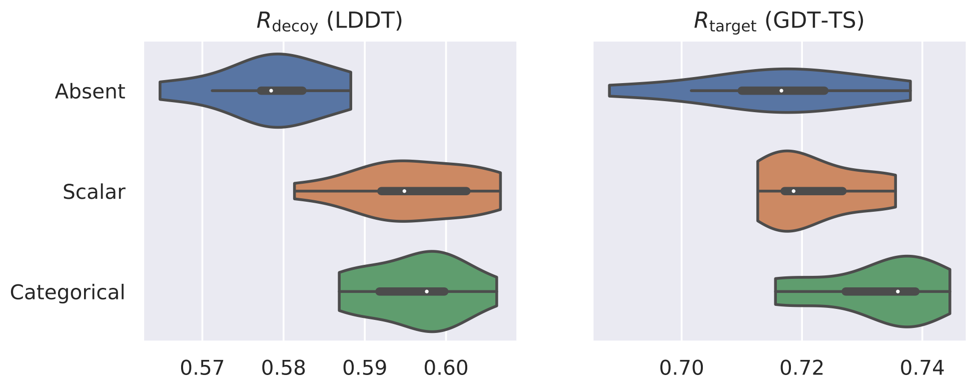

Fig. S5: Sequential separation: absent, encoded as a scalar, encoded as a categorical variable.

S4.2 Transmembrane vs. soluble proteins

In this study, we evaluate how the natural environment of a protein affects the predictive performances of our method. Targets from CASP 11 and 12 are classified as transmembrane and soluble according to Peters *et al.* (2015) and scored separately using GraphQA. Transmembrane proteins behave differently from soluble proteins as a consequence of the environment they are placed in. The former expose non-polar residues to the cellular membrane that surrounds their structure. On the contrary, the latter tend to present polar amino acids to the surrounding water-based solvent.

Since this information is not explicitly provided to the model, we can compare the predictive performances between the two sets and check that it has actually learned a flexible protein representation. The outcome of this evaluation is shown in table S4. While it is evident that GraphQA performs better on soluble proteins, which are more numerous in the training set, it also scores transmembrane proteins to an acceptable degree.

Table S4. Performances of GraphQA on transmembrane and soluble targets from CASP 11 and 12.

	GDT_TS								LDDT		
	FRL	R	R_{target}	RMSE	ρ	ρ_{target}	τ	τ_{target}	R	R_{model}	RMSE
Transmembrane	0.092	0.714	0.538	0.209	0.714	0.463	0.523	0.328	0.679	0.487	0.170
Soluble	0.076	0.790	0.627	0.177	0.796	0.500	0.594	0.361	0.770	0.532	0.155

S5 Additional Metrics

The Quality Assessment literature is rich of metrics to measure the performances of scoring methods. In the main text we tried to keep the exposition uncluttered by only reporting figures for the most important metrics. Here we present a more extensive set of metrics, that further describe our method and can serve as future benchmark.
In the following, we use:

$t = 1, \dots, T$	Target proteins
$d = 1, \dots, D^t$	Decoys of a target
$i, j = 1, \dots, \lvert S^t \rvert$	Residue indexes of a target
$C^{t,d} = \{ (x, y, z)_i \}$	Decoy conformation (tertiary structure)
$C^{t,\text{native}} = \{ (x, y, z)_i \}$	Native conformation (tertiary structure)
$q^{g,t,d} = [\text{GDT\_TS}, \text{GDT\_HA}, \text{TM}, \text{LDDT}, \text{CAD}]$	True global quality scores
$q_i^{\ell,t,d} = [\text{LDDT}_i, \text{CAD}_i]$	True local quality scores
$\text{GraphQA}^g(\mathcal{P}^{t,d}) \in [0, 1]^5$	Predicted global quality scores
$\text{GraphQA}_i^\ell(\mathcal{P}^{t,d}) \in [0, 1]^2$	Predicted local quality scoress

Root Mean Squared Error (RMSE) We compute RMSE between all true and predicted scores.

For local scores, it it the square root of:

$$\text{MSE} = \frac{1}{T} \sum_{t=1}^{T} \frac{1}{D^t} \sum_{d=1}^{D^t} \frac{1}{|S^t|} \sum_{i=1}^{|S^t|} \| q_i^{\ell,t,d} - \text{GraphQA}_i^\ell(\mathcal{P}^{t,d}) \|^2$$

For global scores, it it the square root of:

$$\text{MSE} = \frac{1}{T} \sum_{t=1}^{T} \frac{1}{D^t} \sum_{d=1}^{D^t} \| q^{g,t,d} - \text{GraphQA}^g(\mathcal{P}^{t,d}) \|^2$$

Correlation coefficients (global) We compute the Pearson (R), Spearman (ρ) and Kendall (τ) correlation coefficients between the true and predicted scores *across all targets and decoys*. Since this evaluation does not distinguish between different decoys or between different targets, a high value for these metrics can be misleading. Thus, their *per-model* and *per-target* versions should be also checked.

For each local score:

$$R = \text{Pearson}\left(\{ q_i^{\ell,t,d} \}, \{ \text{GraphQA}_i^\ell(\mathcal{P}^{t,d}) \} \right)$$
$$\rho = \text{Spearman}\left(\{ q_i^{\ell,t,d} \}, \{ \text{GraphQA}_i^\ell(\mathcal{P}^{t,d}) \} \right)$$
$$\tau = \text{Kendall}\left(\{ q_i^{\ell,t,d} \}, \{ \text{GraphQA}_i^\ell(\mathcal{P}^{t,d}) \} \right)$$

For each global score:

$$R = \text{Pearson}\left(\{ q^{g,t,d} \}, \{ \text{GraphQA}^g(\mathcal{P}^{t,d}) \} \right)$$
$$\rho = \text{Spearman}\left(\{ q^{g,t,d} \}, \{ \text{GraphQA}^g(\mathcal{P}^{t,d}) \} \right)$$
$$\tau = \text{Kendall}\left(\{ q^{g,t,d} \}, \{ \text{GraphQA}^g(\mathcal{P}^{t,d}) \} \right)$$

Correlation coefficients per-model For every decoy of every target, we compute the Pearson (R_model), Spearman (ρ_model) and Kendall (τ_model) correlation coefficients between true and predicted residue-level scores (LDDT, CAD). We then report the average correlation coefficients across all decoys of all targets. The *per-model* correlation coefficients estimate the performance of the network to rank individual residues by their quality and distinguish correctly vs. incorrectly folded segments.

Per-model correlation coefficients are computed only for local scores:

$$R_\text{model} = \frac{1}{T} \sum_{t=1}^{T} \frac{1}{D^t} \sum_{d=1}^{D^t} \text{Pearson}\left(\{ q_i^{\ell,t,d} \}, \{ \text{GraphQA}_i^\ell(\mathcal{P}^{t,d}) \} \right)$$

$$\rho_\text{model} = \frac{1}{T} \sum_{t=1}^{T} \frac{1}{D^t} \sum_{d=1}^{D^t} \text{Spearman}\left(\{ q_i^{\ell,t,d} \}, \{ \text{GraphQA}_i^\ell(\mathcal{P}^{t,d}) \} \right)$$

$$\tau_\text{model} = \frac{1}{T} \sum_{t=1}^{T} \frac{1}{D^t} \sum_{d=1}^{D^t} \text{Kendall}\left(\{ q_i^{\ell,t,d} \}, \{ \text{GraphQA}_i^\ell(\mathcal{P}^{t,d}) \} \right)$$

Correlation coefficients per-target For every target, we compute the Pearson (R_{target}), Spearman (ρ_{target}) and Kendall (τ_{target}) correlation coefficients between true and predicted decoy-level scores (GDT_TS, GDT_HA, TM-score, LDDT, CAD). We then report the average correlation coefficients across all targets. With reference to the funnel plots, this would be the correlation between the markers in every plot, averaged across all plots. The *per-target* correlation coefficients estimate the performance of the network to rank the decoys of a target by their quality and select the ones with highest global quality.

Per-target correlation coefficients are computed only for global scores:

$$R_{\text{target}} = \frac{1}{T}\sum_{t=1}^{T} \text{Pearson}\left(\{\, q^{g,t,d} \,\}, \{\, \text{GraphQA}^{g}(\mathcal{P}^{t,d}) \,\}\right)$$

$$\rho_{\text{target}} = \frac{1}{T}\sum_{t=1}^{T} \text{Spearman}\left(\{\, q^{g,t,d} \,\}, \{\, \text{GraphQA}^{g}(\mathcal{P}^{t,d}) \,\}\right)$$

$$\tau_{\text{target}} = \frac{1}{T}\sum_{t=1}^{T} \text{Kendall}\left(\{\, q^{g,t,d} \,\}, \{\, \text{GraphQA}^{g}(\mathcal{P}^{t,d}) \,\}\right)$$

First Rank Loss (FRL) and First Rank Loss top-5 (FRL$_5$) For every target, we compute the difference in ground-truth scores between the true best decoy and the best decoy according to the predicted scores. We then report the average FRL across all targets. This represents the loss in (true) quality we would suffer if we were to choose a decoy according to our ranking. In the funnel plots (figures S6, S7 and S8), FRL can be visualized as the gap between the two vertical lines indicating the true best (green) and predicted best (red).

FRL is only computed for global scores only, for every target t:

$$\text{FRL} = \frac{1}{T}\sum_{t=1}^{T} \left|\max\{\, q^{g,t,d} \,\} - q^{g,t,d^*}\right| \qquad \text{with } d^* = \arg\max_{d} \{\, \text{GraphQA}^{g}(\mathcal{P}^{t,d}) \,\}\,.$$

FRL measures the ability to select a single best decoy for a given target. In our experiments, however, we noticed that FRL is extremely subject to noise, as it only considers top-1 decoys. Thus, we also compute FRL$_5$, which represents the minimum loss across the 5 top-scoring decoys of a target.

***z*-score (z)** For every target, we consider the ground-truth distribution of global scores and compute the z-score of each decoy, relative to the mean and variance of such distribution. For example, for GDT_TS:

$$z^{t,d} = \frac{\text{GDT\_TS}^{t,d} - \text{Mean}\left(\{\, \text{GDT\_TS}^{t,d'} \,\}_{d'=1}^{D^t}\right)}{\text{Var}\left(\{\, \text{GDT\_TS}^{t,d'} \,\}_{d'=1}^{D^t}\right)}$$

Then, we use GraphQA's predictions to identify the best-scoring decoy of each target and average their z-scores:

$$z = \frac{1}{T}\sum_{t=1}^{T} z^{t,d^*} \qquad \text{with } d^* = \arg\max_{d} \{\, \text{GraphQA}^{g}(\mathcal{P}^{t,d}) \,\}\,.$$

Notably, we do not remove outliers and do not set negative z-scores to zero, as commonly done in CASP. The reason is twofold:

- Outlier removal was introduced in CASP4 when the quality of submitted decoys was relatively low. Therefore, a cleanup step was required to ignore the low-quality models that would otherwise skew this metric.
- Negative z-scores are only relevant if some QA method selects a worse-than-average decoy as the best model for a given target, otherwise they are never included in the mean. Since we only compare with top-performing QA methods this step is not necessary.

S6 CASP13 Additional Results

CASP13 is the most recent edition of CASP whose decoy structures are available at the time of writing. Specifically, the inputs of our model, target sequences and predicted tertiary structures can be downloaded from the website. Since native structures have also been released, it is possible to compare our predictions with the ground-truth quality scores, obtained with respect to the target conformations. Furthermore, QA predictions submitted by other participants are available, enabling us to compare results. In this section we present additional results for methods and metrics that are excluded from the main text for sake of brevity.

S6.1 Evaluation of GraphQA

In table S5 we report all metrics computed using our global quality predictions. As indicated, each metric is either: i) computed across all decoys of all targets or ii) first computed on the decoys of each target and then averaged over targets.

Table S5. Evaluation metrics for each of the five global scores predicted by GraphQA. All metrics are computed w.r.t. to the respective ground-truth scores, using the two strategies described in the text: across all targets and average per-target.

	Across all targets					Per-target				
	CAD	GDT_HA	GDT_TS	LDDT	TM-score	CAD	GDT_HA	GDT_TS	LDDT	TM-score
RMSE	0.082	0.107	0.130	0.101	0.130	0.074	0.095	0.119	0.091	0.120
R	0.870	0.846	0.855	0.864	0.864	0.841	0.778	0.779	0.832	0.772
ρ	0.888	0.863	0.856	0.868	0.860	0.809	0.740	0.742	0.805	0.727
τ	0.703	0.669	0.663	0.678	0.669	0.637	0.563	0.563	0.630	0.547
z	/	/	/	/	/	1.508	1.348	1.274	1.312	1.235
FRL	/	/	/	/	/	0.056	0.070	0.081	0.059	0.086
FRL_5	/	/	/	/	/	0.021	0.028	0.030	0.020	0.037

Similarly, in table S6 we report all metrics computed our local quality predictions. These local metrics are either: i) computed across all residues, or ii) computed on the residues of each decoy and then averaged over all decoys of all targets.

Table S6. Evaluation metrics for each of the two local scores predicted by GraphQA. All metrics are computed w.r.t. to the respective ground-truth scores, using the two strategies described in the text: across all decoys of all targets, and average of per-decoy metrics.

	Across all residues		Per-decoy	
	CAD	LDDT	CAD	LDDT
RMSE	0.179	0.145	0.176	0.136
R	0.672	0.804	0.440	0.542
ρ	0.664	0.797	0.423	0.527
τ	0.473	0.599	0.295	0.380
z	/	/	1.093	0.991
FRL	/	/	0.304	0.139
FRL_5	/	/	0.151	0.079

In table S7 we report the 95% confidence intervals for all Pearson correlation coefficients computed for local and global predictions. We apply the Fisher r-to-z method using respectively 12852 pairs of values (global scores) and 3024161 pairs of values (local scores).

Table S7. Confidence intervals at 95% computed using the Fisher r-to-z method. On the left, the Pearson's correlation coefficients R and R_{target} for global predictions. On the right, the Pearson's correlation coefficients R and R_{decoy} for local predictions.

	R	R_{target}
CAD	[0.866, 0.874]	[0.835, 0.846]
GDT_HA	[0.841, 0.851]	[0.771, 0.784]
GDT_TS	[0.851, 0.860]	[0.772, 0.786]
LDDT	[0.860, 0.869]	[0.827, 0.838]
TM-score	[0.859, 0.868]	[0.765, 0.779]

	R	R_{decoy}
CAD	[0.671, 0.673]	[0.439, 0.440]
LDDT	[0.803, 0.804]	[0.542, 0.543]

In table S8 and table S9 we report the z-score and first rank loss of the predicted-best decoy w.r.t. the true best for each target in CASP13, as ranked by the five types of global scores we consider.

Table S8. z-score and FRL loss for global predictions (part 1/2). Each metric is computed between the predicted and groud-truth values for that score type.

	CAD		GDT_HA		GDT_TS		LDDT		TM-score	
	z	FRL	z	FRL	z	FRL	z	FRL	z	FRL
T0949	0.913	0.071	1.249	0.017	1.013	0.006	0.971	0.038	0.866	0.021
T0950	0.583	0.138	1.282	0.083	0.728	0.187	-0.052	0.249	2.419	0.124
T0951	0.494	0.093	0.695	0.017	0.603	0.011	0.570	0.051	0.483	0.012
T0953s1	1.982	0.021	2.281	0.017	1.947	0.014	1.792	0.027	1.989	0.012
T0953s2	2.087	0.045	1.119	0.092	0.920	0.188	1.185	0.097	0.709	0.271
T0954	2.021	0.006	1.596	0.027	1.426	0.024	1.646	0.006	1.189	0.019
T0955	1.360	0.113	0.342	0.305	0.550	0.238	1.159	0.108	0.544	0.295
T0957s1	2.724	0.015	3.383	0.025	2.665	0.051	1.970	0.031	2.247	0.057
T0957s2	0.924	0.109	0.909	0.150	1.020	0.182	1.379	0.072	0.879	0.213
T0958	0.908	0.145	-0.162	0.234	-0.330	0.318	0.115	0.187	-0.597	0.378
T0959	1.909	0.003	1.612	0.000	0.373	0.148	1.456	0.000	0.476	0.113
T0960	1.903	0.059	1.203	0.014	1.100	0.022	1.587	0.093	0.953	0.036
T0961	1.283	0.003	1.181	0.000	1.020	0.000	1.130	0.002	0.795	0.001
T0962	1.361	0.023	1.144	0.061	1.059	0.051	1.073	0.037	0.944	0.042
T0963	1.329	0.070	0.764	0.034	0.797	0.049	0.871	0.157	0.858	0.060
T0964	2.417	0.045	1.992	0.029	1.934	0.032	2.070	0.037	1.559	0.092
T0965	0.990	0.035	0.963	0.003	0.728	0.023	0.934	0.004	0.636	0.025
T0967	0.744	0.145	0.546	0.168	0.559	0.108	0.661	0.107	0.618	0.073
T0968s1	2.564	0.024	2.339	0.062	2.226	0.063	2.257	0.036	2.098	0.087
T0968s2	2.933	0.000	2.265	0.104	2.262	0.102	2.376	0.019	2.266	0.118
T0969	3.176	0.000	3.175	0.000	3.082	0.000	2.869	0.000	2.754	0.000
T0970	1.846	0.050	2.613	0.053	2.204	0.088	1.534	0.088	1.254	0.194
T0971	1.071	0.085	1.120	0.048	0.970	0.015	0.948	0.074	0.525	0.096
T0973	1.121	0.098	0.010	0.289	0.080	0.314	1.005	0.068	0.125	0.296
T0974s1	0.682	0.121	0.508	0.130	0.478	0.112	0.434	0.110	0.528	0.121
T0974s2	1.209	0.017	0.929	0.059	0.533	0.094	0.446	0.153	0.523	0.095
T0975	3.237	0.026	2.832	0.000	2.803	0.000	2.968	0.000	2.639	0.000
T0976	0.244	0.172	0.630	0.090	1.699	0.020	1.428	0.000	1.791	0.011
T0978	1.138	0.051	0.971	0.073	1.352	0.056	1.304	0.057	1.559	0.008
T0979	0.810	0.128	0.672	0.196	0.234	0.277	1.003	0.063	0.295	0.225
T0980s1	0.440	0.107	0.639	0.137	0.504	0.231	0.595	0.138	0.469	0.236
T0980s2	-1.120	0.147	-0.182	0.056	-0.308	0.097	-0.807	0.108	0.447	0.051

Table S9. z-score and FRL loss for global predictions (part 2/2). Each metric is computed between the predicted and groud-truth values for that score type.

	CAD		GDT_HA		GDT_TS		LDDT		TM-score	
	z	FRL	z	FRL	z	FRL	z	FRL	z	FRL
T0982	0.461	0.108	0.055	0.101	0.211	0.175	0.568	0.107	0.398	0.190
T0983	1.115	0.007	0.885	0.005	0.788	0.005	0.938	0.004	0.700	0.002
T0986s1	0.852	0.159	0.217	0.204	0.508	0.209	0.761	0.142	1.968	0.027
T0986s2	1.389	0.082	0.812	0.111	1.516	0.118	1.331	0.093	1.441	0.144
T0987	2.886	0.001	2.278	0.010	2.249	0.013	2.434	0.000	2.572	0.000
T0988	1.925	0.016	1.243	0.081	1.169	0.085	1.326	0.057	1.114	0.065
T0989	0.677	0.096	1.838	0.046	1.897	0.058	1.258	0.101	1.985	0.042
T0991	3.319	0.000	2.857	0.000	4.033	0.000	3.124	0.000	4.399	0.000
T0992	3.002	0.000	2.861	0.000	2.271	0.000	2.528	0.000	2.105	0.000
T0993s1	0.586	0.092	0.806	0.041	0.824	0.010	0.510	0.083	0.651	0.014
T0993s2	0.582	0.043	0.686	0.069	0.539	0.087	0.590	0.058	0.515	0.093
T0995	1.211	0.028	1.044	0.000	0.951	0.002	1.055	0.001	0.770	0.021
T0997	2.493	0.006	2.054	0.004	2.031	0.002	2.090	0.008	1.915	0.005
T0998	-0.006	0.079	1.010	0.074	1.504	0.108	0.850	0.048	1.474	0.132
T1000	2.013	0.022	1.899	0.047	2.048	0.056	1.885	0.037	1.970	0.057
T1001	3.529	0.000	3.246	0.000	2.577	0.000	2.782	0.014	2.237	0.023
T1002	1.934	0.000	1.101	0.021	1.046	0.015	1.571	0.000	0.993	0.020
T1003	0.976	0.032	0.979	0.000	0.835	0.006	0.911	0.011	0.678	0.004
T1004	1.253	0.022	0.788	0.086	0.885	0.057	1.123	0.024	0.927	0.061
T1005	0.547	0.091	0.153	0.142	0.311	0.150	0.525	0.070	0.385	0.122
T1006	0.788	0.066	0.751	0.071	0.670	0.033	0.749	0.054	0.673	0.023
T1008	3.680	0.000	4.669	0.000	3.930	0.000	3.489	0.000	4.067	0.000
T1010	3.205	0.011	3.157	0.037	3.902	0.000	3.469	0.000	3.683	0.000
T1011	1.158	0.006	1.196	0.021	1.110	0.009	0.884	0.038	0.670	0.091
T1013	1.393	0.023	1.467	0.028	1.300	0.016	1.209	0.021	1.074	0.017
T1014	1.140	0.044	0.993	0.021	0.866	0.017	0.927	0.041	0.641	0.041
T1015s1	1.024	0.163	0.961	0.122	0.659	0.204	0.567	0.182	0.708	0.194
T1015s2	1.680	0.012	1.432	0.000	1.130	0.016	1.202	0.008	1.063	0.027
T1016	0.857	0.020	0.829	0.006	0.624	0.011	0.727	0.024	0.499	0.016
T1017s1	1.921	0.000	1.129	0.052	1.053	0.045	1.327	0.016	1.044	0.040
T1017s2	1.132	0.159	0.202	0.266	0.134	0.372	0.295	0.250	0.081	0.436
T1018	1.298	0.004	0.924	0.043	0.812	0.040	1.084	0.011	0.660	0.007
T1019s1	3.464	0.000	2.642	0.073	2.350	0.090	2.710	0.018	2.240	0.152
T1019s2	1.961	0.017	2.231	0.000	1.869	0.000	1.948	0.000	1.773	0.000
T1020	-0.797	0.356	-0.756	0.410	-0.265	0.399	-0.367	0.308	0.251	0.239
T1021s1	1.333	0.045	0.880	0.161	0.977	0.151	1.053	0.066	0.485	0.238
T1021s2	1.427	0.026	1.359	0.000	1.046	0.014	1.159	0.000	0.789	0.046
T1021s3	2.500	0.005	2.079	0.018	1.925	0.021	2.154	0.006	1.721	0.021
T1022s1	1.845	0.034	1.977	0.054	1.905	0.076	1.709	0.091	1.624	0.099
T1022s2	1.552	0.008	1.523	0.052	1.324	0.071	1.168	0.023	1.110	0.068

S6.2 Comparing GraphQA to other quality assessment methods

Where possible, we compute evaluation metrics for all groups who participated in the quality assessment track in CASP13. The metrics for global quality predictions are shown in table S10, and metrics for local quality predictions are shown in table S11. All evaluation metrics are computed inhouse using the official CASP13 submissions from the participating groups. All tables are relative to stage 2 predictions, according to the official categorization. The evaluation code is publicly available.

Decoy-level global quality predictions
For decoy-level predictions, we download the publicly available GDT_TS submissions made by every group for all decoys from CASP13. These predictions are directly comparable with the ground-truth values for GDT_TS computed using open-source tools (appendix S2.1). Furthermore, they correspond to one of the scores that GraphQA is trained to predict at the global level. Therefore, in table S10 we report the evaluation of all metrics described above for GDT_TS predictions. Finally, we produce funnel plots for all targets, where the true and predicted GDT_TS scores are drawn (figures S6, S7, and S8)

Residue-level local quality predictions
For residue-level predictions, comparing GraphQA with other methods is not as straightforward, as GraphQA is trained to predict LDDT and CAD scores, but submissions to CASP require to estimate the distance, in Ångstrom, between the residue in the decoy and its correct position in the native structure using a superposition from GDT_TS. Therefore, we convert our score predictions to "distances" using the following heuristic:

$$\text{dist}_i^{t,d} = \max\left(15,\ 5\sqrt{\frac{1}{\text{GraphQA}_i^{\ell}(\mathcal{P}^{t,d})} - 1}\right). \tag{2}$$

Once the local quality predictions of all groups are expressed as distances, we compare with ground-truth distances downloaded from the CASP portal. In table S11, the entries GraphQA-LDDT and graphqa-CAD represent respectively the LDDT and CAD predictions of GraphQA converted into distance-like values. Notably, some groups are characterized by negative correlation coefficients, which we guess is due to a submission mistake. In fact, those methods might predict a score in the range $[0, 1]$ where 1 represents high quality and might have skipped the conversion to a distance-like value. However, we choose to treat their predictions as submitted and did not apply an extra conversion.

Table S10. Global quality prediction (GDT_TS): performance comparison between all quality assessment groups in CASP13, both single-model and consensus-based. All metrics are computed with respect to ground-truth GDT_TS scores, using the two strategies described in the text: global across targets and average of per-target metrics. For GraphQA, we use the decoy-level output corresponding to GDT_TS. For other QA groups, we use the publicly available submissions from the CASP portal and also report the group ID used in CASP. We consider predictions submitted for stage 2 decoys. Single-model methods are highlighted in bold, to separate them from consensus methods. Rows are sorted by R across all targets.

		**Across all targets**				**Per-target**						
Method		$R\uparrow$	RMSE $\downarrow$	$\rho\uparrow$	$\tau\uparrow$	FRL $\downarrow$	$\text{FRL}_5\downarrow$	$R\uparrow$	RMSE $\downarrow$	z $\uparrow$	$\rho\uparrow$	$\tau\uparrow$
UOSHAN	QA194	.925	.090	.930	.784	.051	.030	.865	.073	1.122	.792	.652
MUfoldQA_T	QA211	.916	.099	.930	.779	.065	.034	.855	.082	1.003	.777	.636
MUFoldQA_M	QA113	.916	.101	.920	.764	.054	.041	.859	.085	1.104	.775	.638
MULTICOM_CLUSTER	QA058	.908	.103	.905	.742	.050	.025	.839	.090	1.112	.767	.609
Davis-EMAconsensusAL	QA171	.903	.145	.904	.742	.062	.039	.856	.127	1.029	.766	.624
Davis-EMAconsensus	QA349	.901	.123	.905	.743	.062	.039	.857	.103	1.015	.766	.625
ModFOLDclust2	QA373	.895	.123	.891	.725	.065	.042	.841	.104	.996	.758	.608
MULTICOM-CONSTRUCT	QA243	.892	.112	.892	.717	.070	.034	.779	.102	.907	.727	.568
**ModFOLD7_cor**	QA213	.890	.110	.888	.720	.104	.041	.735	.091	.569	.665	.514
**ModFOLD7**	QA275	.888	.111	.887	.719	.083	.035	.757	.091	.776	.689	.539
RaptorX-DeepQA	QA334	.883	.159	.905	.736	.070	.039	.762	.138	.925	.708	.556
**ModFOLD7_rank**	QA272	.872	.156	.867	.690	.058	.023	.742	.139	1.063	.688	.526
Wallner	QA457	.858	.200	.887	.711	.077	.036	.708	.177	.874	.644	.494
**GraphQA**	/	.855	.130	.856	.663	.081	.030	.779	.119	1.274	.742	.563
Bhattacharya-ClustQ	QA014	.852	.238	.892	.715	.063	.038	.800	.218	.980	.720	.563
Pcomb	QA083	.847	.239	.899	.729	.080	.036	.759	.218	.838	.679	.526
Pcons	QA022	.835	.277	.885	.711	.086	.050	.718	.258	.788	.652	.502
CPClab	QA471	.820	.143	.837	.652	.084	.033	.656	.132	.822	.655	.492
**FALCON-QA**	QA413	.816	.137	.809	.621	.086	.024	.647	.124	.748	.599	.447
**FaeNNz**	QA027	.803	.141	.788	.602	.080	.032	.661	.134	.865	.627	.471
**ProQ3D**	QA139	.802	.146	.793	.603	.076	.024	.637	.131	.815	.589	.437
MUfoldQA_S2	QA107	.784	.147	.767	.578	.134	.085	.660	.129	.355	.566	.435
**MUFold_server**	QA312	.779	.148	.769	.578	.080	.029	.607	.139	.875	.573	.425
**ProQ3D-CAD**	QA198	.777	.209	.769	.580	.094	.032	.639	.183	.735	.616	.458
**ProQ3D-TM**	QA267	.774	.159	.767	.580	.074	.028	.629	.144	.896	.570	.419
**ProQ3D-lDDT**	QA360	.768	.152	.761	.577	.088	.025	.658	.142	.801	.614	.457
**ProQ3**	QA187	.763	.155	.752	.561	.086	.031	.576	.146	.821	.556	.412
**LamoureuxLab**	QA067	.753	.162	.751	.561	.092	.030	.599	.149	.816	.543	.403
**ProQ2**	QA044	.748	.164	.741	.549	.084	.029	.596	.153	.803	.554	.407
**MESHI**	QA197	.735	.187	.744	.557	.072	.028	.627	.171	.985	.578	.435
**MESHI-corr-server**	QA237	.719	.179	.722	.543	.082	.033	.605	.162	.842	.541	.401
**MESHI-enrich-server**	QA289	.708	.188	.715	.534	.073	.027	.629	.171	.974	.574	.432
**ProQ4**	QA440	.698	.176	.712	.528	.080	.028	.664	.156	.870	.587	.456
**MESHI-server**	QA347	.663	.191	.667	.484	.089	.040	.605	.169	.834	.540	.399
**MASS1**	QA146	.661	.174	.661	.478	.098	.039	.543	.161	.636	.483	.354
**VoroMQA-A**	QA339	.657	.208	.658	.474	.095	.041	.555	.184	.755	.500	.367
**SASHAN**	QA220	.649	.175	.637	.464	.094	.030	.571	.157	.743	.516	.374
**VoroMQA-B**	QA030	.629	.211	.639	.457	.088	.031	.551	.186	.809	.527	.388
MASS2	QA415	.617	.189	.621	.443	.108	.035	.508	.178	.588	.464	.340
**Bhattacharya-Server**	QA102	.603	.240	.593	.419	.107	.033	.477	.223	.569	.475	.340
**Bhattacharya-SingQ**	QA170	.585	.239	.576	.411	.095	.029	.506	.219	.671	.500	.358
**PLU-AngularQA**	QA209	.574	.193	.565	.402	.119	.049	.421	.182	.425	.369	.258
**MULTICOM-NOVEL**	QA023	.534	.272	.548	.380	.089	.039	.495	.229	.739	.454	.328
**3DCNN**	QA359	.478	.205	.543	.382	.121	.058	.490	.191	.535	.451	.326
**SBROD-plus**	QA207	.430	.241	.445	.300	.066	.033	.627	.207	1.049	.575	.419
**SBROD**	QA135	.354	.333	.352	.239	.079	.031	.612	.298	.856	.574	.419
**SBROD-server**	QA364	.352	.244	.357	.241	.074	.030	.599	.213	.916	.558	.408
Kiharalab	QA344	.345	.299	.330	.226	.101	.039	.527	.277	.629	.498	.366
**Grudinin**	QA196	.334	.337	.334	.227	.083	.033	.606	.301	.800	.573	.419
**PLU-TopQA**	QA134	.310	.230	.295	.199	.180	.096	.066	.214	-.152	.055	.038
Jagodzinski-Cao-QA	QA065	.083	.281	.081	.054	.142	.068	.267	.253	.317	.232	.162

Table S11. Local quality prediction (residue-residue distance): performance comparison of all quality assessment groups in CASP13, both single-model and consensus-based. All metrics are computed with respect to ground-truth local distances as computed in CASP. Metrics are averages using the two strategies described in the text: globally across all decoys and average of per-decoy metrics. Since GraphQA predicts LDDT and CAD scores we transform these scores into distances using the heuristic formula described in the text. The QA methods at the bottom of the table that achieve negative correlation coefficients have most likely uploaded score-like values rather than distance-like values in their official submission to CASP. We consider predictions submitted for stage 2 decoys. For each group, we report the group ID used in CASP. Rows are sorted by ρ across all decoys. If ρ is negative we consider its absolute value, since it better reflects the predictive quality of the corresponding method. Single-model methods are highlighted in bold, to separate them from consensus methods.

Method		Across all residues			Per-decoy		
		$R\uparrow$	RMSE $\downarrow$	$\rho\uparrow$	$R\uparrow$	RMSE $\downarrow$	$\rho\uparrow$
UOSHAN	QA194	0.730	3.385	0.751	0.671	3.419	0.622
CPClab	QA471	-0.582	6.536	-0.689	-0.459	6.554	-0.464
Davis-EMAconsensus	QA349	0.518	6.099	0.665	0.698	3.858	0.647
ModFOLDclust2	QA373	0.608	4.171	0.650	0.686	3.409	0.638
**FaeNNz**	QA027	-0.564	6.423	-0.664	-0.400	6.530	-0.396
**ModFOLD7**	QA275	0.599	3.908	0.635	0.603	3.803	0.562
**ModFOLD7_rank**	QA272	0.599	3.908	0.635	0.603	3.803	0.562
Pcomb	QA083	0.595	3.937	0.630	0.571	4.014	0.533
**ProQ3D**	QA139	0.441	4.640	0.625	0.458	4.623	0.438
**GraphQA-LDDT**	/	0.569	4.275	0.624	0.430	4.487	0.410
**ProQ3**	QA187	0.273	16.632	0.618	0.408	11.739	0.447
**ProQ3D-lDDT**	QA360	-0.538	6.479	-0.616	-0.432	6.522	-0.414
**GraphQA-CAD**	/	0.548	5.418	0.613	0.394	5.553	0.384
**ProQ3D-CAD**	QA198	-0.502	6.556	-0.606	-0.367	6.606	-0.376
Yang-Server	QA164	0.653	5.309	0.587	0.671	5.163	0.559
Wallner	QA457	0.541	4.244	0.579	0.541	4.195	0.507
**ProQ4**	QA440	0.515	4.849	0.577	0.409	4.928	0.387
**ModFOLD7_cor**	QA213	0.454	4.353	0.574	0.536	4.344	0.510
**ProQ3D-TM**	QA267	0.468	5.547	0.568	0.461	5.002	0.430
Pcons	QA022	0.519	5.068	0.560	0.543	4.431	0.503
RaptorX-DeepQA	QA334	0.128	35.301	0.550	0.589	7.656	0.546
**LamoureuxLab**	QA067	-0.480	6.454	-0.545	-0.374	6.506	-0.349
**ProQ2**	QA044	0.396	4.716	0.538	0.396	4.770	0.394
**VoroMQA-A**	QA339	0.385	5.325	0.535	0.377	5.354	0.381
**VoroMQA-B**	QA030	0.356	5.629	0.527	0.373	5.461	0.376
**SASHAN**	QA220	0.432	4.467	0.520	0.342	4.649	0.341
**MASS2**	QA415	0.393	4.745	0.494	0.357	4.830	0.354
**MASS1**	QA146	0.392	4.745	0.494	0.356	4.830	0.354
**MULTICOM-NOVEL**	QA023	0.339	4.781	0.380	0.207	5.164	0.192
**3DCNN**	QA359	0.007	59.957	0.372	0.227	6.492	0.252

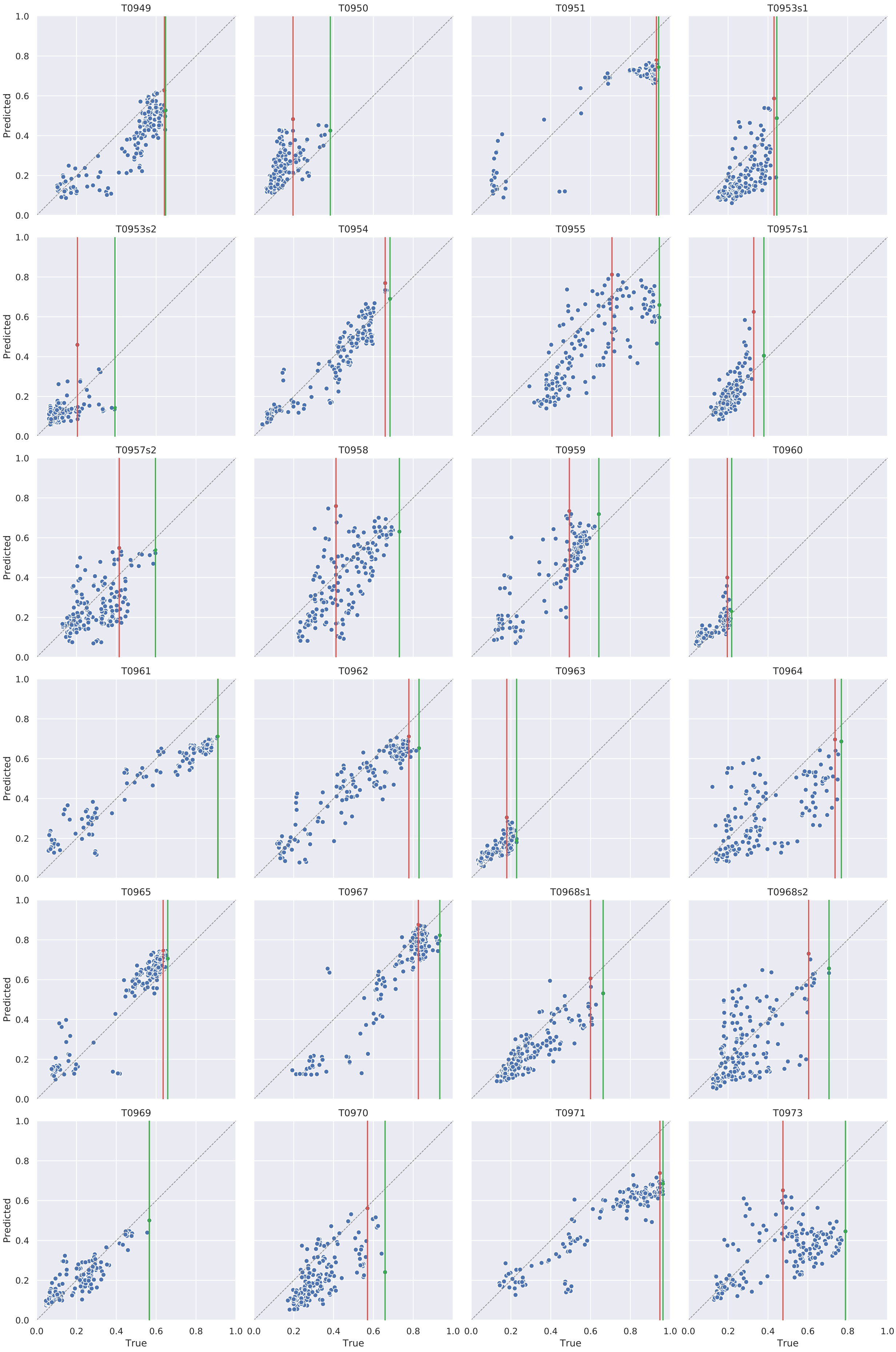

Fig. S6: CASP13: funnels (part 1/3)

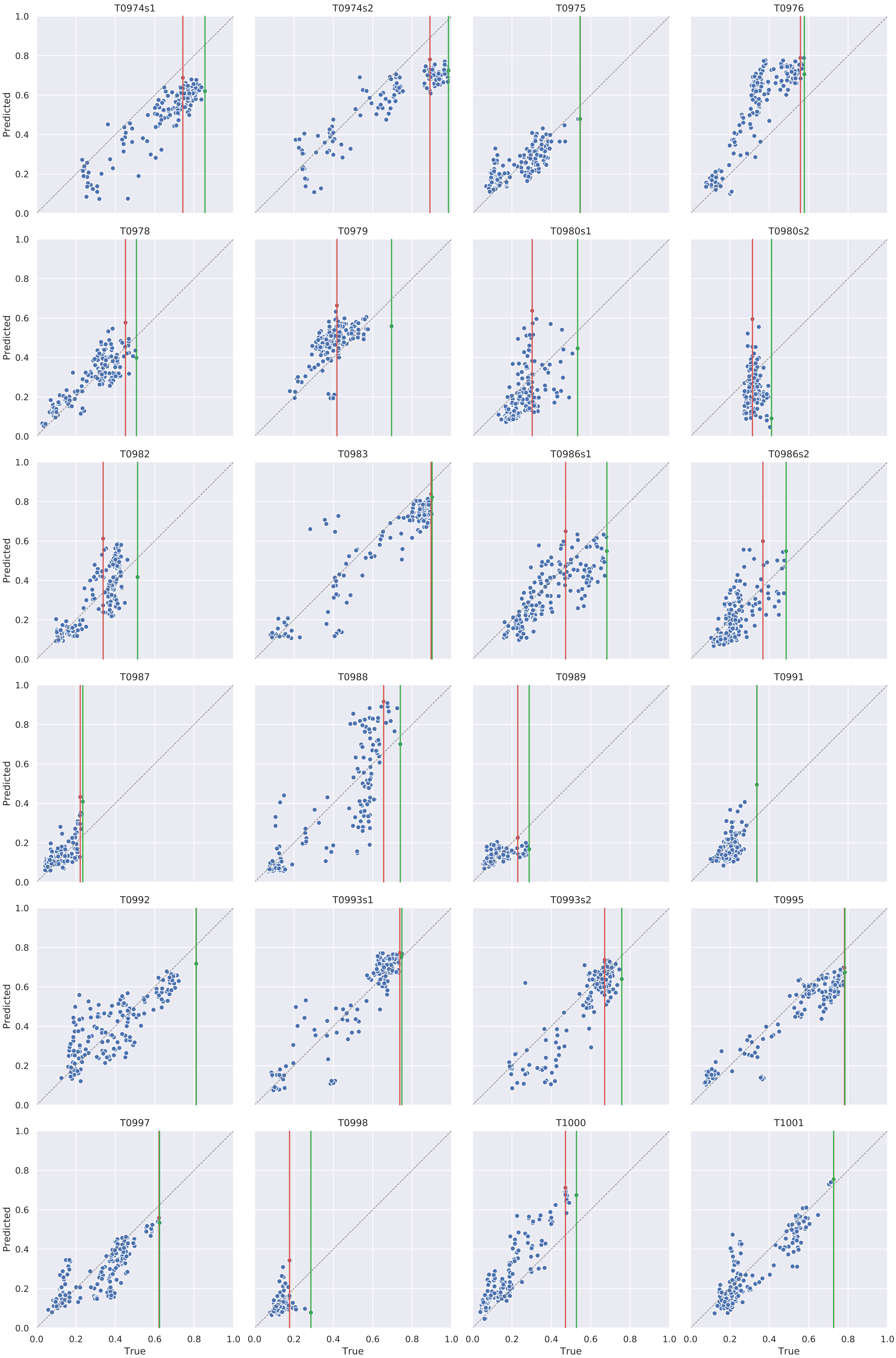

Fig. S7: CASP13: funnels (part 2/3)

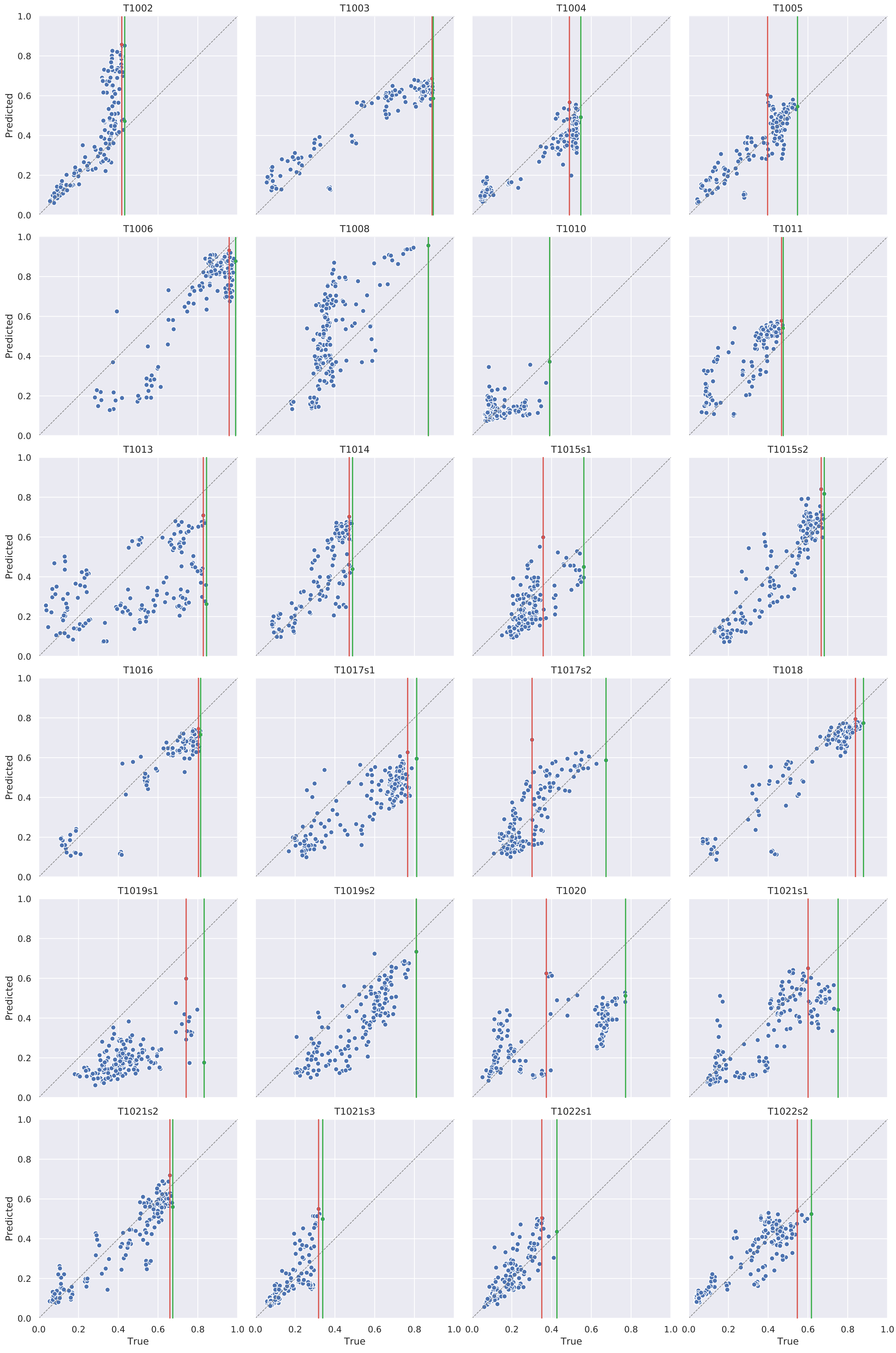

Fig. S8: CASP13: funnels (part 3/3)
